# Liquid fuel generation from algal biomass via a two-step process: effect of feedstocks

**DOI:** 10.1186/s13068-018-1083-2

**Published:** 2018-04-02

**Authors:** Yu-Ping Xu, Pei-Gao Duan, Feng Wang, Qing-Qing Guan

**Affiliations:** 1 College of Chemistry and Chemical Engineering, Department of Energy Chemical Engineering, Henan Polytechnic University, No. 2001, Century Avenue, Jiaozuo, 454003 Henan People’s Republic of China; 2 Faculty of Environmental Science and Engineering, Kunming University of Science and Technology, Kunming, 650500 China

**Keywords:** Algae, Hydrothermal liquefaction, Crude bio-oil, Catalytic upgrading, Tetralin, Upgraded bio-oil

## Abstract

**Background:**

In this study, a two-step processing method (hydrothermal liquefaction followed by catalytic upgrading) was used to produce upgraded bio-oil. A comprehensive screening analysis of algal species, including four microalgae and four macroalgae, was conducted to bridge the gap between previous accounts of microalgae and macroalgae hydrothermal liquefaction and the upgrading process of the resulting crude bio-oils.

**Results:**

Hydrothermal liquefaction using eight algal biomasses was performed at 350 °C for 1 h. The microalgae always produced a higher crude bio-oil yield than the macroalgae due to their high lipid content, among which *Schizochytrium limacinum* provided the maximum crude bio-oil yield of 54.42 wt%. For microalgae, higher amounts of N in the biomass resulted in higher amounts of N in the crude bio-oil; however, contrary results were observed for the macroalgae. The crude bio-oils generated from both the microalgae and macroalgae were characterized as having a high viscosity, total acid number, and heteroatom content, and they were influenced by the biochemical compositions of the feedstocks. Next, all eight-crude bio-oils were treated at 400 °C for 2 h with 10 wt% Ru/C using tetralin as the hydrogen donor. The hydrogen source was provided after tetralin was transformed to naphthalene. All the upgraded bio-oils had higher energy densities and significantly lower N, O, and S contents and viscosities than their corresponding crude bio-oils. However, the H/C molar ratio of the upgraded bio-oils decreased due to the absence of external hydrogen relative to the crude bio-oils. The S content of the upgraded bio-oil produced from upgrading the *Schizochytrium limacinum* crude bio-oil was even close to the 50 ppm requirement of China IV diesel.

**Conclusions:**

Microalgae are better feedstocks than macroalgae for liquid fuel production. Biochemical components have a significant impact on the yield and composition of crude bio-oil. Tetralin does not perform as well as external hydrogen for controlling coke formation. The S content of the upgraded bio-oil can be reduced to 76 ppm for the crude bio-oil produced from *Schizochytrium limacinum*. Upgraded bio-oils have similar properties to those of naphtha and jet fuel.

**Electronic supplementary material:**

The online version of this article (10.1186/s13068-018-1083-2) contains supplementary material, which is available to authorized users.

## Background

With the increasing urgency of mitigating both the energy crisis and environmental pollution, there is an urgent need to find alternative fuel sources that are clean, environmentally friendly, and reproducible. Of these alternative candidates, biomass has emerged as one of the most prospective sources for liquid fuel production due to their high productivity, low pollution, and lack of CO_2_ emission [1, 2]. As an important biomass category, algae, which has adapted to living in aquatic environments (saltwater or freshwater), has emerged as a promising alternative to fossil fuels as it does not compete with edible crops and food and can generate larger amounts of lipid than terrestrial biomass. In addition, the sustained removal of algae will generally benefit the nutrient balance and native ecology of the affected aquatic ecosystems [3]. Therefore, algae present a particularly promising feedstock for future biofuel production.

Thermo-chemical conversion [e.g., hydrothermal liquefaction (HTL) and pyrolysis], in which algae is converted into an energy intensive liquid fuel-crude bio-oil, is one of the most popular routes for algae energy utilization. Algae typically contain very high levels of moisture when harvested, and thus, subjecting such biomass feedstocks to aqueous phase processing is attractive as it can significantly lower the prohibitive energy requirement associated with feedstock drying. HTL can also boost the energy density of the resulting crude bio-oil with respect to pyrolysis oil [4]. To date, many different kinds of aquatic biomasses were tested for crude bio-oil generation, which mainly includes algae (microalgae and macroalgae) [5–9], duckweed [10, 11], water lettuce [12], hydrilla [13, 14], and water hyacinth [15, 16]. In these previous researches, although different algae were employed for HTL, there is no comparability between the results of these experiments because of the different algal species, extraction methods of bio-oil, and definitions of bio-oil yield used. The biochemical compositions of algal biomass can be significantly different, depending on the species, which affects the HTL reactivity. Biller and Ross [14] suggested that biochemical components contributed to bio-oil formation in the following order: lipids > proteins > carbohydrates. In addition, they proposed a simple additive model for predicting the crude bio-oil yield from the biochemical composition. Moreover, although different algae will result in different crude bio-oil yields, all of these crude bio-oils have similar characters, such as poor flowability, high corrosion, low stability, and poor miscibility, as refinery feedstocks at room temperature, and, thus, cannot be used directly without further treatment. In addition, all of these crude bio-oils also have large amounts of heteroatoms such as N, O, and S; and these quantities are significantly higher than the limits defined by ASTM [17]. Therefore, quality improvement of crude algal bio-oil is needed if one expects to use it as a transportation fuel. To date, lots of techniques were tested for the upgrading of crude algal bio-oil, which mainly include solvent addition, emulsification, esterification, supercritical fluidization, hydrotreatment, hydro-cracking, zeolite cracking, and steam reforming [18], among which hydrotreatment is a very promising upgrading process and has a very broad prospect of industrial application in biorefinery. In this process, O, N, and S are eliminated as H_2_O, NH_3_, and H_2_S by a catalytic hydrogenation reaction with added hydrogen. In addition, solvents, such as ethanol [19], methanol [20], and water [21–24], have been employed for crude algal bio-oil upgrading to reduce formation of carbonaceous deposits, extend the catalyst lifetime, and improve mass and heat transfer limitations. However, the alcohols tend to react or decompose and need to be isolated from product fractions after the hydrotreating process, which will increase cost of the liquid fuel. Moreover, these solvents are rich in oxygen, which not only acted as reaction media, but also as reactants during the hydrotreating process. Therefore, they would react with bio-oil and increase oxygen level of the bio-oil during the hydrotreating process. Alternatively, tetralin can overcome the shortcomings of the oxygenated solvents, which also can increase the yield of desirable products and reduce the formation of carbon deposition [25]. Therefore, tetralin might be a promising solvent for the upgrading of crude algal bio-oil.

To date, vast majority studies have concentrated on upgrading crude bio-oils produced from HTL of microalgae [21–24] and duckweed [26–28]. However, upgrading crude bio-oils generated from other algal feedstocks, rather than algae and duckweed, has not been reported. Under identical reaction conditions, crude bio-oils with different molecular components might show different reaction activities. However, until now, a side-by-side parallel comparison of the yields and physicochemical characteristics of upgraded bio-oils arising from upgrading of crude bio-oils that were yielded from the HTL of different algae under identical conditions has not been conducted. Taking into account the current research focused on liquid fuel production from algae and the rapid development in the field of crude bio-oil upgrading, more databases that link the molecular composition of crude bio-oil to the yield and properties of upgraded bio-oil are still needed.

Therefore, in the present study, we first provide a comparative assessment of the yields and qualities of crude bio-oils generated from eight different algae under identical conditions. Next, all of the crude bio-oils were subsequently subjected upgrading under identical conditions to examine the effect of crude bio-oil type on the yields and qualities of the resulting upgraded bio-oils. Tetralin was employed as a solvent and hydrogen donor instead of using subcritical or supercritical water as extensive work on upgrading crude algal bio-oil in subcritical or supercritical water has already been conducted [21–24]. To the authors’ knowledge, the previous investigation on processing a range of crude bio-oils produced from different algae in tetralin has not been conducted. This gap in the literature motivated the present study. New fields have been opened up by this research in three aspects. We use a larger suite of algal biomass feedstocks (eight different materials) than has been used in any prior study, we consider a two-step processing approach wherein hydrothermal liquefaction precedes catalytic upgrading, and we used tetralin instead of water as the upgrading medium. The S level of the upgraded bio-oil was even close to the 50 ppm requirement of China IV diesel.

## Methods

### Materials

Eight algae, including four microalgae (*Nannochloropsis oceanica:* NO, *Auxenochlorella pyrenoidosa*: AuP, *Arthrospira platensis*: ArP, and *Schizochytrium limacinum*: SL) and four macroalgae [*Ulva prolifera*: UP, *Saccharina japonica* (*Areschoug*): SJ, *Lemna minor* (duckweed): LM, and *Pyropia yezoensis:* PY] were used. All these algae were dried in an oven at 110 °C for 12 h and pulverized into particles (≤ 100 mesh) prior to analysis and use. Freshly deionized water (DI) was employed for all experiments. Table 1 provides the proximate and ultimate analyses of these algae.

**Table 1:** Proximate and ultimate analyses (wt%, dry basis) of algal biomass feedstocks

	NO	AuP	ArP	SL	UP	SJ	LM	PY
Ash	10.69	5.46	12.62	7.85	34.24	28.10	20.51	12.45
Volatiles	72.05	72.37	63.46	70.75	43.43	53.27	56.95	62.02
Fixed carbon	17.26	22.16	23.92	21.40	22.33	18.63	22.54	25.53
Lipid	11.30	12.70	6.20	28.60	3.20	2.70	2.22	0.56
Protein	41.69	53.56	62.69	46.88	27.50	14.44	28.13	36.19
Carbohydrate	36.32	28.28	18.49	18.29	35.06	54.76	49.16	50.80
C	50.10	52.07	44.16	53.99	30.04	32.24	38.08	40.55
H	7.45	7.15	6.27	7.53	4.66	4.71	4.87	6.18
N	6.67	8.57	10.03	7.50	4.40	2.31	4.50	5.79
O	32.92	25.00	21.65	20.58	30.35	19.78	20.72	29.40
S	0.76	0.62	0.64	0.56	1.73	0.64	0.52	2.03
HHV (MJ/kg)	22.61	23.84	20.21	25.40	12.22	14.20	16.44	18.25
Na	4.26	1.03	2.64	3.08	8.85	5.08	0.49	2.63
Mg	2.65	0.35	1.41	0.45	0.57	0.29	0.66	0.12
Al	0.11	0.15	0.34	0.14		1.11	0.30	1.08
Si	1.79	0.79	4.39	1.79	0.42	3.63	2.05	1.24
P	0.10	0.45	2.49	0.18	0.73	1.71	1.27	1.63
Cl	1.07	2.44	0.62	0.44	19.90	4.92	4.23	1.58
K	2.51	0.38	4.64	2.73		4.52	6.62	2.15
Ca	1.61	3.92	0.87	0.99	2.31	5.70	8.92	3.09
Ti	0.01		0.01		0.02		0.04	
Cr		0.02			0.01		0.01	
Mn	0.03	0.06	0.01	0.01	0.34	0.01	0.17	
Fe	0.28	0.08	0.73	0.67	0.21	3.50	0.77	1.51
Ni					0.06		0.08	
Cu	0.02	0.03	0.02			0.01	0.08	0.01
Zn	0.01		0.01	0.01	0.02	0.20	0.02	0.06
Br		0.04			0.39		0.02	
Sr		0.18	0.01		0.64		0.09	
Mo		0.01			0.03		0.03	
Ru							0.03	

Ru/C (5 wt% Ru, surface area = 966 m^2^/g, metal dispersion = 23.2, and average particle size = 25 μm) was commercially available from Zhengzhou Alfachem Co., Ltd., and employed as the upgrading catalyst. Ru/C was directly mixed with crude bio-oil without undergoing prereduction under H_2_ prior to use. Tetralin and dichloromethane (Shanghai Aladdin Biochemical Technology Co., Ltd., Shanghai, China) with a purity ≥ 99.9% were used as the hydrogen donor solvent and extraction solvent, respectively. Argon and helium (Changzhou Jinghua Industrial Gas Co., Ltd., Changzhou, China) were obtained with purities of 99.999%.

Two custom-made high-pressure and corrosion-resistant batch reactors (Zhengxin Instrument Factory, Yancheng, Jiangsu, China) were used to perform the experiments. The total internal volumes of these two reactors are 100 mL for the HTL reactions and 58 mL for the upgrading reactions, respectively. These two reactors were treated with supercritical water at 400 °C for 4 h to eliminate any lubricating oil during their manufacture and to oxidize the reactor wall for reduction of possible catalysis effect of the reactor wall. A custom-built molten-salt bath that consists of KNO_3_ and NaNO_3_ at a mass ratio of 5:4 was employed as a heat source to heat these two reactors.

### HTL

The previous studies [3–5] suggested that the maximum bio-oil yield was typically obtained at 350 °C for 1 h when subjected algal biomass to the HTL process. Therefore, 350 °C and 1 h were selected as parameters for the HTL reactions. Aliquots of 20.0 g of dry algae powder and 40.0 mL of DI water were added into the 100 mL reactor. The air in the loaded reactor was eliminated by flushing the reactor head space with helium for roughly 20 min and further charged with helium at 0.01 MPa. This known amount of helium acted as an internal standard to determine the gas yield. The loaded and pressurized reactor was put into a molten-salt bath pretreated to 350 °C and maintained at 350 ± 2 °C using a temperature controller. About 18 min was cost as the temperature inside the reactor achieved at 350 °C, and the reaction time was set zero at this time. The reaction lasted for 60 min. The reactor was taken out of the molten-salt bath and immediately placed into a cool water bath to quench the reaction. The cooled reactor was placed at room temperature for 4 h to ensure the uniformity of gas in the reactor.

The outlet of the reactor was connected to a six-way valve inlet of gas chromatography, and the pressurized gases in the reactor were fed into a 1 mL sample loop as the reactor valve was cutoff slowly until carrier gas in the sample loop was completely exchanged with the sample gas. After analyzing the gas fraction, the reactor was opened to recover the liquid fraction. The gas weight was estimated by mass difference before and after exhausting the gas from the reactor. The reactor was charged with 20 mL dichloromethane and sealed and vigorously shaken by hand. The same operation was repeated for three times to maximize recovery of reactor contents. The solid residue in the reactor contents was isolated by filtration and dried in an oven at 110 °C for 12 h and then weighed. The separated liquid phase was further subjected to a separatory funnel to get aqueous phase and dichloromethane extracts. The dichloromethane was then evaporated to get the crude bio-oil. The yield of each product fraction was calculated as its mass divided by the mass of algae loaded into the reactor.

### Hydro-upgrading

A previous research suggested that 400 °C was a more appropriate temperature for crude algal bio-oil upgrading [7]. Thus, in the present study, 400 °C was employed as the upgrading temperature. The mentioned above 58 mL batch reactor was used to perform the upgrading experiments. In a typical run, 5.0 g of crude bio-oil, 0.5 g of the Ru/C catalyst, and 15 g of tetralin were introduced into the reactor and tightly sealed. The air in the loaded reactor was removed by purging the reactor head space with helium for about 20 min and further charged with helium at 1.0 MPa. No additional hydrogen was added. The upgrading reaction was initiated by immerging the reactor body into a molten-salts tank preheated to 400 °C. Approximately 30 min elapsed as the temperature inside the reactor mounted from room temperature to 400 °C, and then, the reaction time was counted. After the upgrading reaction was lasted for 2 h, the reactor was taken out of the molten-salt tank and placed into an ice-water bath to stop the reaction. The same procedure for subsequent product separation as that of the HTL mentioned above was employed. Dichloromethane was also used as an extraction solvent. The noncatalyst solid residue was considered as the coke whose amount was calculated from the mass difference between the solid residue and the initial loaded catalyst. The mass ratio of each product fraction to the total mass of the loaded crude bio-oils and tetralin was defined as the yield of each product fraction.

The results presented in this study are all the average results of three repeated experiments. The results reported in this study represent the mean values for three independent trials. The uncertainties are reported as the experimentally determined standard deviations.

### Product analysis

Bligh–Dyer method was used to quantify the crude fat content in algae [29]. The ash content was tested according to the ASTM [30]. The volatile matter content was quantified from the weight difference before and after heating a known amount of algae sample at 575 °C under a N_2_ atmosphere. The fixed carbon content was calculated by the following equation: ${\text{fixed carbon}}\% \, = \, 100\% \, - \,{\text{moisture}}\% \, - \,{\text{volatile}}\% \, - \,{\text{ash}}\% .$ Kjeldahl method [31] was applied to roughly estimate the protein content in algae. The carbohydrate content was calculated by the following equation: ${\text{carbohydrate}}\% \, = \, 100\% \, - \,{\text{moisture}}\% \, - \,{\text{crude fat}}\% \, - \,{\text{protein}}\% .$ X-ray fluorescence (Bruker S8 TIGER, Karlsruhe, Germany) was used to analyze the inorganic composition of algae and Table 1 presents the results.

An organic element analyzer (Flash 2000) (Thermo Fisher Scientific, Waltham, MA, USA) was used to quantify C, H, O, N, and S in the sample. The total S and N contents in the bio-oil were determined based on ASTM D5453-12 [32] and D4629-12 [33], respectively, as their values in the bio-oil are below detection limit of the organic element analyzer. Dulong formula was employed to estimate the higher heating value (HHV) of the algae and bio-oils as follows:

$$ {\text{HHV (MJ/Kg)}}\, = \,0. 3 3 8{\text{C}} + 1.428\;\left( {\text{H}\, - \,{\text{O}}/ 8} \right)\, + \,0.0 9 5{\text{S}},$$

where *C*, *H*, *S*, and *O* represent the weight percentage of carbon, hydrogen, sulfur, and oxygen in a sample, respectively.

A gas chromatography coupled with a mass spectrometry (7890A GC-5975C) (Agilent Technologies Co. Ltd., CA, Palo Alto, USA) was used to qualify molecular compositions of the bio-oil. More details are available in Ref. [21]. An automatic total-acidity determination instrument (YUS-A2 Zhengzhou Zhonggu Machinery Equipment Co., Ltd., China) was used to test the total acid number (TAN) of the bio-oil. Thermogravimetric analysis was employed to simulate the boiling-point distribution of the bio-oil. An SDT Q600 Simultaneous DSC-TGA instrument (TA Instruments Co. Ltd., New Castle, DE, USA) was used. A known amount of sample was heated to 780 °C at a heating rate of 10 °C/min in N_2_ atmosphere at a flow rate of 10 mL/min.

## Results and discussion

### Characterization of the algal biomass feedstocks

Table 1 shows the proximate and ultimate analyses of the algal biomass feedstocks used in the present study. The relative amount of crude fat, which contributes most significantly to the bio-oil production during the thermo-chemical process, was significantly higher in the microalgae than that in the macroalgae. The macroalgae contain higher amount of ash than the microalgae. Therefore, as expected, macroalgae contain less volatile than microalgae. The fixed carbon ranged from 17.26 to 23.92% for the microalgae and from 18.63 to 25.53% for the macroalgae. Based on differences in lipid, volatile matter, and fixed carbon, the yields of the HTL products can be expected to be different between microalgae and macroalgae. The differences in the mineral content of algae also depend on the geographical location, oceanic residence time, season, and the species of algae. The microalgae were usually cultured in fresh water, while the macroalgae were cultured in seawater. Therefore, the macroalgae (particular the UP) contained higher chlorine contents (1.58–19.90 wt%) than the microalgae because of their saline environment. Both the microalgae and macroalgae contained large amounts of macrominerals (Ca, K, Mg, Si, Al, P, and Na) and trace elements (Mo, Ti, Fe, Cr, Cu, Mn, Sr, Ni, Zn, and Ru). Biosorption might be responsible for the presence of trace elements in algae. Slagging and fouling usually happened during the thermo-chemical process of those biomasses that contain high amount of alkali metals. The product distribution during the HTL was also influenced by the ash contained in the biomass.

### TG analysis of the algal biomass feedstocks

Detailed and accurate characterization of algae is a necessity for any biomass-to-biofuel conversion process. Understanding how the individual component of biomass and reaction products interacts at each stage in the process is important for researchers.

**Fig. 1:**
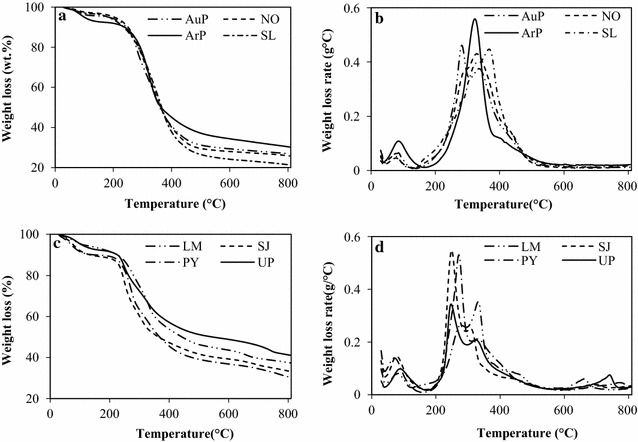
**a** TG curves for microalga AuP, NO, ArP, and SL; **b** DTG curves for microalga for AuP, NO, ArP, and S; **c** TG curves for microalga LM, SJ, PY, and UP; **d** DTG curves for microalga for LM, SJ, PY, and UP

The thermal behavior of algae can be learned by subjecting it to a TG analysis under an inert atmosphere. Both TG and derivative thermogravimetric (DTG) analyses of the algae samples were performed by checking the mass loss with increasing time and temperature. Figure 1a–d shows all the results. This is the first study to compare the thermal decomposition behavior of a range of different algae under identical conditions. Algae mainly consist of proteins, carbohydrates, and lipids, among which the lipid content of microalgae is commonly higher than that of macroalgae. These components were transformed into bio-oil, gas, solid residue, and water-soluble products during HTL. The TG and DTG curves can be used to determine the temperature at which each biochemical component of algae becomes completely pyrolyzed. Of course, secondary reactions between biochemical compounds can also affect the yield of the product distribution. In general, the microalgae species showed similar TGA and DTG curves that were different from those of the macroalgae species. The TG curve revealed three stages of thermal degradation for the algae during pyrolysis. As the DTG curves shown in Fig. 1b, d, Stage I is the dehydration step at temperatures ranging from 30 to 150 °C during which the cellular water in the feedstock evaporated. Mass loss was observed for the both microalgae and macroalgae, indicating that the physically dried algae still contained some water. More water mass loss was observed for the macroalgae than the microalgae due to the higher carbohydrate content in the macroalgae. As the temperature rose to 200 °C, the mass loss entered the second stage, devolatilization, during which the crude lipids, proteins, and carbohydrates gradually decomposed, leading to a significant weight reduction and the production of main pyrolytic products. In Stage II, two shoulders were observed in the DTG curves that corresponded to the initial decomposition temperatures of the proteins and carbohydrates [34], respectively. In Stage III, within the temperature range of 500–800 °C, more volatiles were released and a char residue was formed. Since the macroalgae contained more ash (mainly consists of inorganic salts) than the microalgae, more weight loss was also observed for the macroalgae when the temperature rose by more than 600 °C due to the vaporization of the salts. The DTG curves of all algae indicated that 350 °C was the suitable temperature. Therefore, 350 °C was chosen as the HTL temperature for the subsequent study.

### Effect of algal biomass feedstocks on the yield of the crude bio-oil and solid residue

**Fig. 2:**
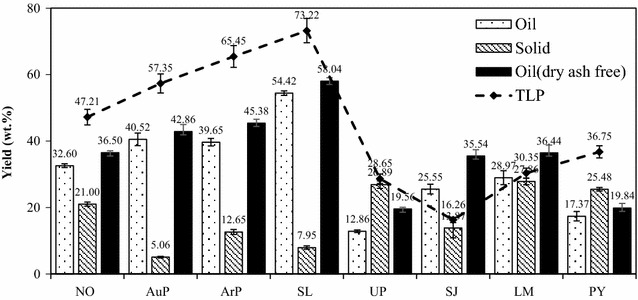
Effect of algal biomass feedstocks on the yield of the crude bio-oil and solid residue (350 °C, 60 min)

**Fig. 3:**
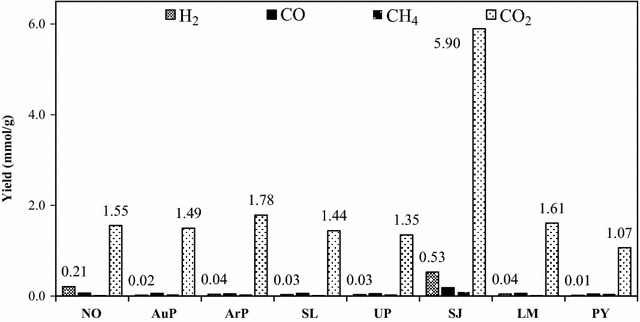
Effects of algal biomass feedstocks on the yield of gaseous products (350 °C, 60 min)

HTL can convert algae to crude bio-oil, solid residue, water-soluble products, and gases. In this present study, only the yield of the solid residue, crude bio-oil, and gaseous products was concerned. Figure 2 reveals the effects of the different algae on yields of the crude bio-oil and solid residue produced from HTL at 350 °C for 60 min. The yields of the bio-oil and solid residue were estimated based on the dry feedstocks. For comparison purpose, the bio-oil yield was also calculated based on the dry-ash free feedstocks. Figure 2 indicates that the crude bio-oil and crude bio-oil (dry-ash free, DAF) yields derived from microalgae were all larger than those of produced from macroalgae. The decomposition of any of the biochemical components, such as the proteins, lipids, and carbohydrates, in the feedstock could dedicate to the production of the crude bio-oil, among which carbohydrate decomposition mainly resulted in the formation of water-soluble products [14]. Therefore, the product yields and total lipid and protein content (TLP) value are also listed in Fig. 2. For microalgae, a clear trend was observed, that is, the larger the TLP content was, the larger the crude bio-oil yield_(DAF)_ was. For macroalgae, however, the crude bio-oil yield_(DAF)_ showed no clear trend. Table 1 shows that the macroalgae commonly contained a certain amount of ash that would affect the HTL process and, subsequently, the product distribution. Of course, this catalytic role was likely due to the different reactivities of the individual lipids, proteins, and carbohydrates. The crude bio-oil yields also showed significant differences within the microalgae or macroalgae species. For the microalgae, the crude bio-oi yield_(DAF)_ ranged from 36.50 to 58.04 wt%. For the macroalgae, the crude bio-oi yield_(DAF)_ ranged from 19.56 to 36.44 wt%. The compositions of various microalgae or macroalgae species generally exhibit pronounced seasonal variation. In addition, such species can be strongly affected by the temperature, geographical location [35], water salinity, and aqueous nutrient content [36]; hence, samples from the same species that are grown in alternative climates can differ substantially. For the NO, a 43 wt% crude bio-oil yield was obtained at 350 °C for 60 min by Brown et al. [5]. They used a feedstock concentration of 4.5 wt% compared to the 33 wt% feedstock used in the present study. Zou et al. [37] suggested that increasing the concentration of feedstock would cause a reduced bio-oil yield. Normally, a higher bio-oil yield was observed at a lower feedstock concentration; however, a large amount of water will be required at the same time, which will increase energy consumption and costs for handling the down-stream waste water. Zhou et al. [38] achieved a crude bio-oil yield of 23 wt% as they hydrothermally liquefied UP at 300 °C for 30 min with 5 wt% of added Na_2_CO_3_. Therefore, in view of the crude bio-oil yield, SL is the algal species with the greatest potential for the generation of crude bio-oil. Figure 2 also indicates that the solid residue yields produced from macroalgae are overall higher those obtained using microalgae; this result is consistent with the higher ash and carbohydrate contents from the macroalgae as carbohydrates are more difficult to convert than proteins and lipids [32]. HTL of the algal biomass would also produce a certain amount of gaseous products which mainly consisted of H_2_, CO, CH_4_, and CO_2_ together with a small quantity of C_2_–C_5_ hydrocarbons. Figure 3 presents the amounts of gaseous products produced from the HTL of the eight algae. The yield of CO_2_ was always the largest, which is consistent with the previous studies on the HTL of biomass [6, 9]. Two reactions such as water–gas shift and steam reforming are responsible for the formation of CO_2_. In addition, the decarboxylation of fatty acids also favored the production of CO_2_. Among the examined eight algae, SJ produced the highest CO_2_ yield of 5.9 mmol/g. H_2_ can also be generated during steam reforming and water–gas shift reactions; however, the H_2_ yield was much lower than that of the CO_2_, indicating that the production mechanisms of these two products were different. SJ provided the highest H_2_ yield of 0.53 mmol/g. CO was always present in the lowest amount in the gaseous fraction as the initial formed CO would be consumed from reactions such as the water–gas shift and methanation as the reaction proceeds [6]. The production of CH_4_ mainly occurred during the methanation reaction, and its yield was low for all algal biomass feedstocks.

### Characterization of the crude bio-oil and solid residue

Table 2A, B presents the elemental compositions of the crude bio-oils and solid residues produced from the eight algae subjected to HTL. These two tables also provide the TAN, H/C molar ratio, HHV, and energy recovery (ER). The ER was the ratio of the total energy in the crude bio-oil to the total energy in the feedstock.

**Table 2:** Elemental composition (wt%), TAN, and other properties of crude bio-oils produced from the HTL of different algal biomass feedstocks and elemental composition (wt%) of solid residue produced from the HTL of different algal biomass feedstocks

Biomass	TAN	C	H	N	O	S	H/C	HHV (MJ/kg)	ER
A									
NO	56.73	73.79	9.65	5.77	6.21	0.64	1.57	37.67	0.54
AuP	58.78	74.34	9.62	6.62	7.07	0.73	1.55	37.67	0.64
ArP	57.60	73.89	9.65	7.56	6.77	0.98	1.57	37.64	0.74
SL	59.85	74.23	9.21	5.85	7.48	0.39	1.49	36.94	0.79
UP	75.36	76.40	9.03	6.10	8.25	0.92	1.42	37.33	0.39
SJ	37.82	74.91	8.57	4.15	8.99	0.40	1.37	35.99	0.75
LM	59.02	73.90	8.67	6.54	7.11	0.48	1.41	36.14	0.64
PY	87.83	74.21	8.50	6.98	9.64	1.81	1.37	35.67	0.34

	NO	AuP	ArP	SL	UP	SJ	LM	PY
B								
C	22.82	13.54	6.93	54.75	34.70	31.57	28.11	62.54
N	0.82	1.94	1.07	1.68	3.17	1.38	2.34	6.18
H	3.25	1.85	1.18	9.12	2.90	1.89	2.24	4.39
S	0.03	0.16	0.24	0.27	1.91	0.85	0.63	1.17

The heteroatoms likely evidenced the existence of the compounds derived from proteins, carbohydrates, and lipids in the algae and formed in reactions, such as depolymerization, decomposition, and addition reactions [36]. Table 2A shows that the C and H contents and energy densities of all crude bio-oils are significantly larger than those of their feedstocks; these results are consistent with those from the previous studies [4–16]. All the crude bio-oils showed similar C content that ranged from 73.79 to 76.40 wt%. The H content of the microalgae was larger than that of the macroalgae for all samples. Consequently, the H content in the crude bio-oils produced from the microalgae was higher than that produced from the macroalgae for all samples. However, within the microalgae or macroalgae groups, the feedstocks with higher hydrogen content did not produce a crude bio-oil with a higher hydrogen content. The O content of all crude bio-oils was significantly lower than that of their corresponding feedstocks, indicating in situ deoxygenation occurred simultaneously during HTL. This deoxygenation process could increase the energy density of the crude bio-oils and thus improve the properties of the crude bio-oils. Compared with the feedstocks, the N content in the crude bio-oils produced from the microalgae was generally reduced. However, contrary results were observed for the macroalgae and these results were also evidenced by Zhou et al. [34]. Hence, it appears to be more difficult to remove N from macroalgae compared to microalgae. The N in the crude bio-oil was mainly derived from the conversion of proteins; the larger the protein content in the algal biomass was, the larger the N in the crude bio-oil was. The S content in all crude bio-oils was generally reduced relative to the feedstock, but this reduction was small. The H/C molar ratio is an important indicator of the quality of a bio-oil; a higher H/C molar ratio indicates a higher quality bio-oil. The crude bio-oils produced from the microalgae have higher H/C molar ratios than those produced from the macroalgae. The energy density of the crude bio-oils showed little variation across the species, ranging from 36.94 to 37.67 MJ/kg for the microalgae and from 35.67 to 37.33 MJ/kg for the macroalgae, despite the significant variation in the feedstock biochemical compositions and crude bio-oil yields. Due to the higher yield and energy density of the crude bio-oil produced from the microalgae, a higher ER was also observed for the microalgae, among which SL presented the highest ER of 0.79. The total ER from the crude bio-oil, solid residue, and gas indicated that a massive energy loss occurred in the aqueous phase, particular for the macroalgae. The lost energy could be recovered using additional processing steps such as hydrothermal gasification. The TAN values of all crude bio-oils produced from the microalgae were similar to each other and ranged from 56.73 to 59.85. The TAN values of all crude bio-oils produced from the macroalgae were different from each other and ranged from 37.82 to 87.83, indicating the much different biochemical composition of the macroalgal crude oil. All the crude bio-oils produced either from the microalgae or macroalgae contained heteroatom concentrations that were significantly higher than the requirement of ASTM [39]. Therefore, further treatment is needed before considering the utilization of algal bio-oil. Although the crude bio-oils were obtained by the solvent extraction method, small amounts of impurities would still reside in the crude bio-oils and might interfere with the catalytic activity of the catalyst during the subsequent upgrading process.

Table 2B shows that C is the predominant element in all the solid residues, indicating that some of the organic matter in the algal biomass was not completely converted during the HTL, particularly the carbohydrates. The solid residue produced from PY contained the highest C content of 62.54 wt%, indicating that the organic matter in PY was the most difficult to convert. In contrast, the solid residue produced from ArP contained the lowest C content of 6.97 wt%, indicating that the organic matter in ArP was the easiest to convert. All the solid residues contained a certain amount of N and S, implying that some protein or protein derivatives resided in the solid residue.

**Fig. 4:**
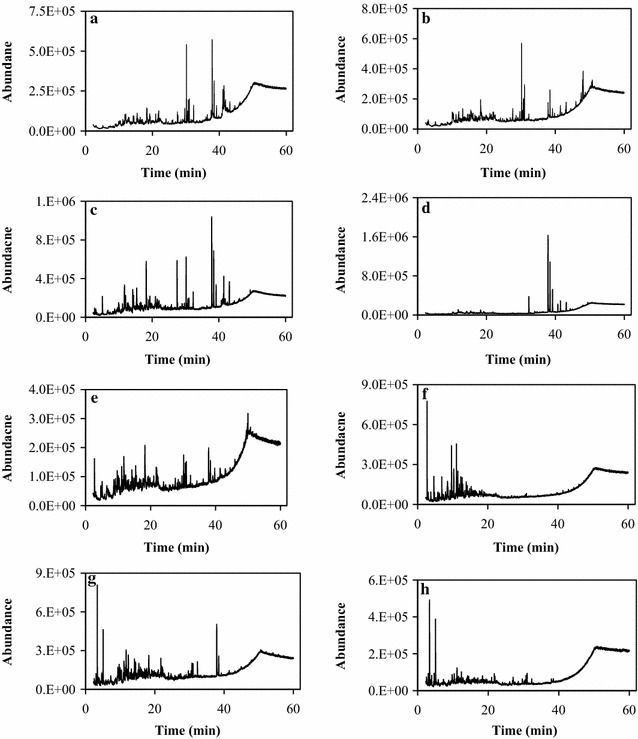
**a** Total ion chromatograms of crude bio-oil produced from NO; **b** Total ion chromatograms of crude bio-oil produced from AuP; **c** Total ion chromatograms of crude bio-oil produced from SL; **d** Total ion chromatograms of crude bio-oil produced from ArP; **e** Total ion chromatograms of crude bio-oil produced from UP; **f** Total ion chromatograms of crude bio-oil produced from SJ; **g** Total ion chromatograms of crude bio-oil produced from LM; **h** Total ion chromatograms of crude bio-oil produced from PY

To compare the differences among the molecular compositions of the crude bio-oils generated from the eight different algae, the individual components in the crude bio-oils were qualified by the aid of GC–MS. The temperature of the gasification chamber was set to 300 °C. The thermogravimetric analysis (TG) suggested that a minimum of 50–60 wt% of the material in the crude bio-oil samples was volatilized at this temperature, whereas the rest exhibited a higher boiling point. Because the bio-oils did not totally volatilize, the acquired data are relevant only to the volatile fraction and do not necessarily represent all the ingredients of the bio-oil. Figure 4a–h shows the total ion chromatograms (TICs) of the crude bio-oils derived from those eight different algae. Significant TIC differences in the crude bio-oils are clearly observed in Fig. 4a–h. In particular, clear differences exist between the crude bio-oils derived from the microalgae and macroalgae because of the different biochemical compositions among the algae. Only a few large peaks at retention times shorter than 40 min appeared for all of the crude bio-oils, among which the TICs of the crude bio-oils are centered within 20 min for the macroalgae and 20–40 min for the microalgae. That is, the crude bio-oils produced from macroalgae contained more light-end products than those of produced from microalgae. Computer matching and a mass spectral library were used to facilitate compound identification. Additional file 1: Table S1 summarizes the GS–MS identification of the major components in the crude bio-oils derived from the eight different algae. For all crude bio-oils, only those compounds whose area % in the TIC was over 1.0% were provided. For the microalgae, NO, AuP, and ArP produced crude bio-oils with a certain amount of phytane and phytene, but the crude bio-oils produced from AuP, SL, and ArP also contained significant quantities of palmitamide, among which that from ArP also contained a certain amount of pyrrolidinone derivatives. In contrast, benzene derivatives were the major products of the crude bio-oils produced from macroalgae. For example, the crude bio-oil produced from UP contained 17.4% benzene and 14.8% ethyl benzene, but the crude bio-oil produced from SJ contained 9.3% 2,3-dimethyl-2-cyclopenten-1-one. The GC–MS results suggested that biomasses with different biochemical components would lead to crude bio-oils with different molecular compositions.

The boiling-point distribution of all crude bio-oils was determined by the TG analysis. A known amount of bio-oil sample was heated to 780 °C at a heating rate of 10 °C/min in N_2_ atmosphere at a flow rate of 10 mL/min. Slight thermal cracking, which would increase the ratio of light-end fractions, was likely at severe temperatures. Table 3 lists the boiling-point distribution of the crude bio-oils determined using the TG, indicating that all of the crude bio-oils contained high-molecular-weight components with boiling points between 150 and 450 °C; this result agrees with the high viscosity of all crude bio-oils. For all the crude bio-oils, slight weight losses, less than 0.3%, below 35 °C were observed. These weight losses corresponded to the removal of residual dichloromethane in the bio-oil. Increasing the temperature would results in an increase of the weight loss. At a temperature range of 35–150 °C, the weight losses of the crude bio-oils produced from macroalgae, particularly from the UP and PY, were greater than those in the crude bio-oils produced from microalgae. The same oil loss trend was observed at a temperature range of 150–250 °C. However, at a temperature range of 250–350 °C, the weight losses of the crude bio-oils derived from microalgae were larger than those of the crude bio-oils derived from macroalgae. The weight losses of the crude bio-oils derived from either microalgae or macroalgae basically became equivalent after further increasing the temperature. The TG analysis suggested that the crude bio-oils derived from macroalgae contained higher low-boiling-point fractions relative to the crude bio-oils produced from microalgae, and severe treatment is needed to use these crude bio-oils as transportation fuels.

**Table 3:** Thermogravimetric analysis of crude bio-oils produced from the HTL of different algal biomass feedstocks

Distillate range (°C)	≤ 35	35–150	150–250	250–350	350–450	≥ 450
NO	0.26	9.38	29.90	32.05	19.80	8.61
AuP	0.08	7.51	28.90	34.09	13.51	15.91
ArP	0.14	7.67	34.90	32.35	13.97	10.97
SL	0.24	6.85	26.99	39.80	16.75	9.37
UP	0.28	12.51	32.10	27.78	12.83	14.50
SJ	0.19	9.77	34.25	27.50	16.90	11.39
LM	0.14	9.54	31.02	26.18	13.66	19.46
PY	0.70	17.20	34.30	24.37	10.64	12.79

### Effect of the crude bio-oils on the yields of the upgraded bio-oil and coke

The goal of the upgrading process is to reduce the viscosity and decrease the N, O, and S contents of crude bio-oils. The crude bio-oil was converted into three major fractions of upgraded bio-oil, solid residue, and gaseous products after it was subjected to treatment with tetralin and Ru/C at 400 °C for 2 h. The mass ratio of the crude bio-oil to tetralin was 1:3. This present study mainly centered on the effects of the crude bio-oils on the yields of the upgraded bio-oil, coke, and gaseous products.

**Fig. 5:**
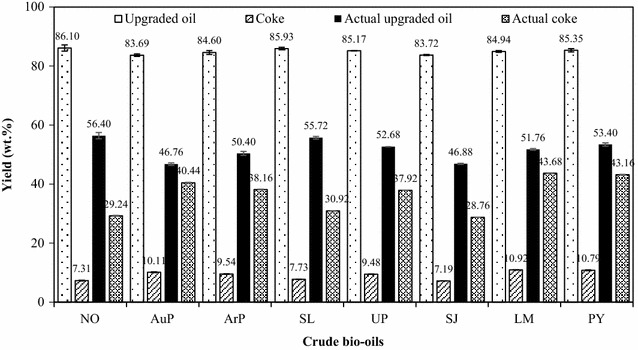
Effect of crude bio-oils on the yield of upgraded bio-oil and coke resulting from catalytic upgrading of crude bio-oils at 400 °C for 2 h

Figure 5 shows the effects of crude bio-oils on the yields of the upgraded bio-oil and coke. A blank experiment with only tetralin (without the addition of crude bio-oil) was also performed under the same reaction conditions. The results show that 96 wt% liquid products were recovered and almost no coke was observed, suggesting that the tetralin was only slightly decomposed. Therefore, the upgraded bio-oil predominantly consisted of tetralin and its derivatives. For comparison purposes, the actual upgraded bio-oil and coke yields are also provided in Fig. 5. The yields of the upgraded bio-oil and the actual upgraded bio-oil are reported within a lower range as mass loss occurred during the sample handling process, particularly during the solvent elimination process. As shown in Fig. 5, the crude bio-oils only slightly affected the yield of the upgraded bio-oil due to the existence of a large amount of tetralin in the products, which ranged from 83.69 to 96.10 wt% for microalgae and 83.72 to 85.35 wt% for macroalgae, respectively. However, the crude bio-oil affected the actual yield of the upgraded bio-oil due to the large differences among the molecular compositions of the crude bio-oils. Due to the high viscosity and asphaltene content of the crude bio-oils, depolymerization and dehydrogenation generally occurred at severe temperatures during the upgrading process [21, 22], and coke was subsequently formed. Since only a minimal amount of coke was formed during the blank experiment, the coke yield was dominantly produced from the crude bio-oil. Figure 5 shows that the type of crude bio-oil used affected the coke yield during the crude bio-oil upgrading, particular the actual coke yield. Table 3 indicates that the heavy-oil fractions with boiling points > 450 °C in the crude bio-oil produced from ArP and AuP were higher than 10 wt%, particularly for the crude bio-oil produced from AuP (15.91 wt%). Therefore, higher coke yields were observed for those crude bio-oils produced from ArP and AuP, 10.11 and 9.54 wt%, which corresponded to actual coke yields of 40.44 and 38.16 wt%, respectively. The same coke yield trends were also observed for the crude bio-oils produced from macroalgae. A 17.3 wt% coke yield was observed as the pretreated AuP bio-oil was hydrothermally treated at 400 °C for 4 h with Ru/C and 6 MPa H_2_ [22], and a 21.8 wt% coke yield was obtained when the pretreated AuP bio-oil was thermally treated at 400 °C for 1 h with Pt/γ-Al_2_O_3_ and 6 MPa H_2_ [21]. In the present study, the actual coke yield could reach as high as 40.44 wt%, indicating that tetralin is not as effective as external hydrogen for suppressing coke formation or that the hydrogen provided was not sufficient to control the coke formation. These cokes would cover the catalyst surfaces during the upgrading process or enter the pores of the catalyst supports and decrease or deactivate the catalyst activity. Therefore, effective coke control is indispensable for maintaining the high activity of catalysts during crude bio-oil upgrading.

The crude algal bio-oils upgrading also produced some gaseous products. The main identified gaseous products were H_2_, CO, and CH_4_. A similar set of gaseous products was reported as the crude bio-oil was treated in supercritical water [21, 22]. Only small quantities of CO_2_ were obtained as the O in the crude bio-oils was eliminated in the form of H_2_O and not from decarboxylation.

### Characterization of the upgraded bio-oils and coke

Table 4 lists the elemental compositions, HHVs, TAN, and ER of the upgraded bio-oils obtained from the upgrading process at 400 °C for 2 h in tetralin with Ru/C. The S and N contents of all upgraded bio-oils were below the detection limit of the common elemental analyzer and quantified according to ASTM D5453-12 [32] and D4629-12 [33], respectively. In all cases, the quality of each upgraded bio-oil exceeded that of the original crude bio-oil from the HTL of algae, because the C content and HHV increased and the N, S, and O contents and TAN decreased. Reactions such as denitrogenation, desulfurization, and deoxygenation contributed to the reduction in the heteroatom content and the corresponding increase in the carbon content after catalytic upgrading. The presence of tetralin during the upgrading process could dilute the crude bio-oils and possibly reduce the production of coke relative to the crude bio-oil alone. Tetralin could also act as a hydrogen donor to promote denitrogenation, deoxygenation, and desulfurization. Moreover, the tetralin could also dilute the N and S and decrease the possibility of catalyst damage. Since the C content of tetralin is 90.0 wt% and tetralin was recovered together with the upgraded bio-oil, and thus, all of the upgraded bio-oils had a higher C content than those of their original crude bio-oils. The H contents of all upgraded bio-oils decreased relative to those of the crude algal bio-oils due to the hydrogen consumption during N, O, and S removal in terms of NH_3_, H_2_S, and H_2_O, respectively. Therefore, external hydrogen is needed if one expects to obtain an upgraded bio-oil with a high H content. GC–MS analysis indicated that a large proportion of tetralin changed to naphthalene as it contributed its hydrogen to the crude bio-oil hydrogenation reaction. Compared with that of the original crude bio-oils, the O content of the upgraded bio-oils was remarkably decreased as severe deoxygenation occurred during the upgrading process. Severe deoxygenation also reduced the TAN from 37.82 to 87.83 for the crude bio-oils to 1.02–3.62 for the upgraded bio-oils. The N content and, in particular, the S content of the upgraded bio-oils obtained in this study were the lowest in the upgraded bio-oils obtained from upgrading the crude algal bio-oils in the presence of Ru/C [21, 22]. The S content of the upgraded bio-oil produced from upgrading the SL crude bio-oil was even close to the requirement of China IV diesel of 50 ppm. The H/C molar ratio of tetralin was 1.20 and the H/C molar ratio of the crude algal bio-oils varied from 1.37 to 1.57. However, the H/C molar ratios of the upgraded bio-oils varied from 1.06 to 1.20, again indicating that hydrogen was consumed during the upgrading process. The crude bio-oils had a minimal influence on the HHVs of the upgraded bio-oils due to the high proportion of tetralin and its derivatives, which ranged from 40.54 to 42.29 MJ/kg. These calorific values are very close to those reported in the previous literatures [21, 22]. Since the yield and HHVs of the upgraded bio-oils were similar to each other, the crude bio-oils had a minimal effect on the ER, which varied from 0.81 to 0.86. The energy loss was mainly ascribed to the mass loss from the products during sample handling.

**Table 4:** Elemental composition (wt%), TAN, and other properties of upgraded bio-oils arising from the upgrading of crude bio-oils produced from the HTL of different algal biomass feedstocks and elemental composition (wt%) of solid residue produced after upgrading of the crude bio-oils produced from the HTL of different algal biomass feedstocks

	TAN	C	H	N (mg/L)	S (mg/L)	O^a^	H/C	HHV (MJ/kg)	ER
A									
NO	1.02	88.22	8.76	3536	91	0.24	1.19	42.29	0.86
AuP	1.33	85.46	8.53	4260	119	0.18	1.20	41.05	0.81
ArP	0.68	86.77	8.30	4906	200	0.43	1.15	41.12	0.82
SL	0.96	87.39	7.74	4097	76	0.32	1.06	40.54	0.83
UP	3.52	87.21	8.55	4526	188	0.47	1.18	41.62	0.84
SJ	1.57	87.26	8.25	3893	97	0.40	1.13	41.21	0.82
LM	3.62	85.91	8.24	4457	115	0.32	1.15	40.76	0.83
PY	1.83	87.18	8.48	4281	216	0.48	1.17	41.51	0.85

	NO	AuP	ArP	SL	UP	SJ	LM	PY
B								
C	66.32	71.56	63.99	77.92	66.43	74.38	54.26	61.85
N	3.58	4.50	5.11	2.50	4.21	3.22	5.55	3.93
H	2.40	3.04	3.20	2.89	3.07	2.93	3.24	2.70
S	0.35	0.40	0.55	0.36	0.47	0.38	0.43	1.52

^a^Calculated by difference

Since the catalyst could not be isolated from the solid residue after the reaction, the solid residue (catalyst + coke) was directly analyzed using the elemental analyzer. Table 4B lists the elemental compositions of all solid resides. Since the coke and catalyst stayed together after the upgrading process, these values present only the averaged elemental compositions of the coke and catalyst. Table 4B shows that C (higher than 50 wt%) was the predominant element with Ru/C present in the solid residue. A certain amount of H was also detected in the solid residue, and the amount of H ranged from 2.40 wt% for NS to 3.20 wt% for ArP, indicating the existence of organic matter in the solid residue. Large amounts of S and N were also observed in the solid residue, indicating that Ru/C could adsorb N- and S-containing compounds or N- and S-containing polymers to form coke. Basically, the higher the N and S contents of the crude bio-oils were, the higher the N and S contents of the solid residues were. These N and S atoms could poison the active sites and deactivate the catalyst.

The individual components in the upgraded bio-oils were qualified by the aid of GC–MS. Additional file 2: Figure S1 shows the representative TICs for the upgraded bio**-**oils dissolved in dichloromethane. The compound identification was carried out by employing a computer matching and a mass spectral library. Table 5 presents the identities of the major individual molecular components in the upgraded bio**-**oils with maximum matching rate to the mass spectra library. Because of the large differences in the crude algal bio-oils, the upgraded bio-oils also exhibited different TICs. The major peaks that appeared in the range of 9–25 min show the derivatives of tetralin and that the dehydrogenation product (naphthalene) was the major product, indicating that the hydrogen sources for the hydrodenitrogenation, hydrodesulfurization, and hydrodeoxygenation reactions mainly originated from the dehydrogenation of tetralin. The upgrading reaction parameters employed in the present study removed some of the fatty acids that typically contain in the crude bio-oils and decreased the levels of N- and S-containing compounds. The upgraded bio-oils contain much higher levels of alkane products than the crude bio-oils. Pentadecane (21.91 min) presented the highest amount in the upgraded bio-oils, but other alkanes ranging from C9 to C30 were also existed. Additional file 2: Figure S1 also shows that the upgraded bio-oils produced from upgrading the microalgal bio-oil contained higher concentrations of alkanes than those produced from upgrading the macroalgal bio-oil. The compound distributions of the upgraded bio-oils are similar to those from the previous publications [21, 22].

**Table 5:** Tentative identities and area % of major peaks in total ion chromatograms for different upgraded bio-oils

RT (min)	Compound name	NO	AuP	ArP	SL	UP	SJ	LM	PY
9.952	Naphthalene, decahydro-	0.27	0.31		0.29	0.31	0.32	0.31	0.31
10.094	Benzene, butyl-	0.40	0.40		0.37	0.31	0.59	0.46	0.34
10.922	Indane, 1-methyl-	1.84	2.01		1.70	1.75	2.60	2.70	1.79
11.207	Naphthalene, decahydro-, cis-	0.30	0.29		0.31	0.26	0.24	0.26	0.27
13.135	Naphthalene, 1,2,3,4-tetrahydro-	69.12	69.23		73.65	72.57	64.66	64.16	72.08
13.736	Naphthalene	22.45	21.66		18.89	22.99	28.60	28.45	22.62
17.301	Naphthalene, 1-methyl-		0.24				0.44	0.50	
21.913	Pentadecane	0.48	0.47		1.19	0.29	0.22	0.30	0.29
27.476	Heptadecane		0.32				0.11		
30.180	Hexadecane, 2,6,10,14-tetramethyl-		0.25						
32.308	Pentadecanenitrile		0.55		1.56				0.23
36.415	Octadecanenitrile		0.41						

TGA was used to provide an estimation of the distillation range distribution of the upgraded bio-oils. Table 6 lists the boiling-point distributions of the upgraded bio-oils as test by the TG analysis. For all upgraded bio-oils, a small amount of weight loss was observed at ≤ 35 °C, indicating the presence of residual dichloromethane. Compared with the original crude bio-oils, the vast majority of the weight loss of all upgraded bio-oils appeared in the range of 35–250 °C; this behavior resembles to that of naphtha and jet fuel. Severe temperatures or/and the addition of accelerated the cracking reactions during the upgrading process, changing the molecular composition from heavy macromolecules to light-end products. More specifically, the upgraded bio-oils produced from microalgal bio-oil contained more boiling fractions between 35 and 150 °C, while the upgraded bio-oils produced from macroalgal bio-oil contained more boiling fractions between 150 and 250 °C.

**Table 6:** Thermogravimetric analysis of different upgraded bio-oils

Distillate range (°C)	≤ 35	35–150	150–250	250–350	350–450	≥ 450
NO	0.50	65.90	29.15	1.60	0.24	2.61
AuP	0.43	56.89	37.60	1.54	0.20	3.34
ArP	0.37	42.59	51.81	1.94	0.36	2.93
SL	0.22	46.03	48.60	1.63	0.31	3.21
UP	0.21	41.64	49.00	4.33	1.04	3.78
SJ	0.37	29.76	61.64	4.42	1.53	2.28
LM	0.23	41.99	48.65	4.39	1.06	3.68
PY	0.26	45.65	48.29	3.60	0.75	1.46

## Conclusion

Both microalgae and macroalgae could be converted into crude bio-oils using HTL at 350 °C for 60 min. The production of the crude bio-oils mainly involved the decomposition of lipids and proteins. Microalgae produced higher crude bio-oil yields than macroalgae. The feedstock biochemical components significantly affected the yield and quality of the crude bio-oils. Obvious deoxygenation reactions happened during HTL. The N in the microalgae was more difficult to remove than that of in the macroalgae during HTL. The TANs of all crude bio-oils produced from microalgae were similar to each other, but major differences were found in the crude bio-oils produced from macroalgae. Phytane, phytene, palmitamide, and pyrrolidinone derivatives were the major products in the crude bio-oil produced from microalgae, while benzene and its derivatives were the major products in the crude bio-oils produced from macroalgae. The TG analysis indicated that all of the crude bio-oils contained high-molecular-weight components with boiling points between 150 and 450 °C.

Upgrading decreased the viscosity and decreased the N, O, and S contents of the crude bio-oils. The crude bio-oils only slightly affected the yield of the upgraded bio-oils. The coke yield was dominantly produced from the crude bio-oil. The higher the high boiling-point fractions of the crude bio-oil were, the higher the coke yield was. Tetralin was not as effective as external hydrogen for suppressing coke formation. The H contents of all upgraded bio-oils decreased relative to those of the crude algal bio-oils due to the hydrogen consumption during N, O, and S removal in term of NH_3_, H_2_S, and H_2_O, respectively. Therefore, external hydrogen is needed if one expects to obtain an upgraded bio-oil with a high H content. Severe deoxygenation reduced the TAN from 37.82 to 87.83 for the crude bio-oils to 1.02–3.62 for the upgraded bio-oils. The S level in the upgraded bio-oil produced from upgrading the SL crude bio-oil was even close to the requirement of China IV diesel of 50 ppm. The upgrading reaction parameters employed in the present study removed some of the fatty acids that typically contain in the crude bio-oils and decreased the levels of N- and S-containing compounds. Compared with the original crude bio-oils, the vast majority weight loss of all upgraded bio-oils appeared in the range of 35–250 °C; this behavior is similar to the properties of naphtha and jet fuel.

## Additional files

**Additional file 1: Table S1.** Tentative identities and area % of the major peaks in the total ion chromatograms for bio-oils produced from eight different algal biomasses.

**Additional file 2: Figure S1.** Total ion chromatograms of upgraded bio-oils produced from the upgrading of eight different crude bio-oils.

## Abbreviations

HTL: hydrothermal liquefaction
ASTM: American Society for Testing and Materials
NO: *Nannochloropsis oceanica*
AuP: *Auxenochlorella pyrenoidosa*
ArP: *Arthrospira platensis*
SL: *Schizochytrium limacinum*
UP: *Ulva prolifera*
SJ: *Saccharina japonica*
LM: *Lemna minor* (duckweed)
PY: *Pyropia yezoensis*
TAN: total acid number
TG: thermogravimetric
DTG: derivative thermogravimetric
DAF: dry-ash free
HHV: higher heating value
ER: energy recovery
TICs: total ion chromatograms
