# Supplementary material for: Liquid fuel generation from algal biomass via a two-step process: effect of feedstocks
Source: Biotechnol Biofuels. 2018 Apr 2;11:83. doi: 10.1186/s13068-018-1083-2 (PMC5879921; doi:10.1186/s13068-018-1083-2)
Supplement: Supplementary file 1 — Additional file 1: Table S1. Tentative identities and area % of the major peaks in the total ion chromatograms for bio-oils produced from eight different algal biomasses. [file 13068_2018_1083_MOESM1_ESM.doc]

**Table S1.** Tentative identities and area % of major peaks in total ion chromatograms for bio-oils produced from eight different algae

| T(min) | Compound name | AuP | NO | ArP | SL | LM | SJ | PY | UP |
| --- | --- | --- | --- | --- | --- | --- | --- | --- | --- |
| 2.746 | n-Propyl acetate | a |  |  |  | 3.40 | 7.02 |  |  |
| 3.011 | Methyl isobutyl ketone |  |  |  |  |  |  |  | 2.72 |
| 3.108 | Disulfide, dimethyl |  |  |  |  |  |  |  | 1.11 |
| 3.160 | 2,3-Dimethyl-1,4-pentadiene |  |  |  |  |  |  |  | 2.28 |
| 3.361 | Toluene |  |  |  |  |  |  | 4.86 | 17.40 |
| 3.755 | 2H-Pyran, 3,4-dihydro- |  |  |  |  |  | 1.31 |  |  |
| 3.801 | Furan, 2,3-dihydro-4-methyl- |  |  |  |  |  |  |  | 2.30 |
| 4.001 | 1,4-Hexadiene, 2,3-dimethyl- |  |  |  |  |  |  |  | 1.38 |
| 4.525 | 2,4-Octadiene |  |  |  |  |  |  |  | 1.42 |
| 4.680 | Cyclopentanone, 2-methyl- |  |  |  |  | 1.10 | 2.53 |  | 3.42 |
| 4.784 | (R)-(+)-3-Methylcyclopentanone |  |  |  |  |  | 1.53 |  |  |
| 5.133 | Ethylbenzene |  | 1.06 | 1.52 |  | 1.27 |  | 2.72 | 14.80 |
| 5.528 | Cyclopentanone, 2,5-dimethyl- |  |  |  |  |  | 1.29 |  |  |
| 5.799 | Cyclooctane |  |  |  |  |  |  |  |  |
| 5.838 | 1,2-Cyclopentanedione, 3-methyl- |  |  |  |  |  |  |  | 1.50 |
| 6.213 | 2-Cyclopenten-1-one, 2-methyl- |  |  |  |  |  | 1.18 |  |  |
| 6.563 | Pyrimidine, 4,6-dimethyl- |  |  |  |  | 3.71 |  |  |  |
| 6.873 | Cyclopentanone, 2-ethyl- |  |  |  |  |  | 2.53 |  |  |
| 8.413 | 2,4-Hexadiene, 3,4-dimethyl-, (Z,Z)- |  |  |  |  |  | 1.51 |  |  |
| 8.523 | 6-Methyl-3-heptyne |  |  |  |  |  | 3.36 |  |  |
| 8.620 | Pyrazine, 2-ethyl-6-methyl- |  |  |  |  | 4.79 | 1.49 |  |  |
| 9.202 | Benzene, 1-methyl-3-(1-methylethyl)- |  |  |  |  | 1.37 |  |  |  |
| 9.247 | 1,3-Hexadiene, 3-ethyl-2-methyl-, (Z)- |  |  |  |  |  | 1.74 |  |  |
| 9.312 | Phenol |  |  |  |  |  |  | 1.05 |  |
| 9.325 | 4-Pyridinamine |  |  |  |  |  |  | 2.05 |  |
| 9.448 | Furan, 2,4-dimethyl- |  |  |  |  |  | 1.43 |  |  |
| 9.745 | 2-Cyclopenten-1-one, 2,3-dimethyl- |  |  |  |  | 3.30 | 9.31 | 2.14 | 3.79 |
| 10.056 | 2-Pyrrolidinone, 1-methyl- |  | 7.97 | 2.30 | 1.16 | 3.91 |  | 3.32 |  |
| 10.140 | Furfuryl hexanoate |  |  |  |  |  | 1.81 |  |  |
| 10.366 | 2-Cyclopenten-1-one, 2,3,4-trimethyl- |  | 1.55 |  |  | 6.08 | 12.68 |  | 4.06 |
| 10.929 | Phenol, 3-methyl- |  |  |  |  |  |  | 1.03 |  |
| 10.936 | 1-Phenyl-1-butene |  |  |  |  |  |  |  | 1.40 |
| 11.091 | Ethanone, 1-(1-cyclohexen-1-yl)- |  |  |  |  |  |  | 2.68 |  |
| 11.117 | 1-Acetamide, N-(3-aminophenyl)- |  |  | 1.35 |  |  |  |  |  |
| 11.149 | Ethanone, 1-(1-cyclohexen-1-yl)- |  |  |  |  |  |  |  | 7.23 |
| 11.446 | 3-Fluoro-o-xylene |  |  |  |  |  | 3.19 |  |  |
| 11.511 | Pyrazine, 2-methoxy-3-methyl- |  |  |  |  |  |  |  | 1.47 |
| 11.563 | 2-Cyclopenten-1-one, 2,3,4,5-tetramethyl- |  |  |  |  |  | 1.05 |  |  |
| 11.621 | 1H-Pyrrole, 3-ethyl-2,4-dimethyl- |  |  |  |  |  |  | 2.27 | 1.97 |
| 11.699 | Quinuclidine-3-ol |  |  | 5.09 |  |  |  |  |  |
| 11.705 | 1H-Imidazole, 1-methyl-4-nitro- |  |  |  |  |  |  | 4.29 |  |
| 11.757 | 1-Ethyl-2-pyrrolidinone |  | 2.07 |  |  |  | 1.74 |  |  |
| 11.783 | Glutarimide | 1.43 |  |  |  |  |  |  |  |
| 11.796 | 2-Methyl-1-ethylpyrrolidine |  |  |  | 3.53 |  |  |  |  |
| 11.880 | Phenol, 4-methyl- | 1.39 |  |  |  | 6.47 |  |  |  |
| 11.932 | 1,3-Benzenediamine, 4-methoxy- |  |  |  |  |  | 2.14 |  |  |
| 12.016 | 2,5-Pyrrolidinedione, 1-methyl- |  |  | 1.47 |  |  |  |  |  |
| 12.042 | 2,6-Piperidinedione, 4,4-dimethyl- |  |  |  |  | 1.18 |  |  |  |
| 12.055 | Cycloheptylamine | 1.35 |  |  |  |  |  |  |  |
| 12.300 | 4-Butyl-6-ethyl-5-methyl-2H-pyran-2-one |  |  |  |  |  | 2.00 |  |  |
| 12.346 | Pyrimidine, 2-methoxy-5-methyl- |  |  |  |  | 2.12 |  |  |  |
| 12.352 | 1H-Pyrrole, 2,3,4,5-tetramethyl- |  |  |  |  |  | 2.35 | 2.89 | 4.52 |
| 12.559 | Acetamide, N-cyclopentyl- |  |  | 1.48 |  |  |  |  |  |
| 12.682 | 2-Cyclopenten-1-one, 2,3,4,5-tetramethyl- |  |  |  |  |  | 1.87 |  |  |
| 12.792 | Formaldehyde, dimethylhydrazone |  |  | 1.74 |  |  |  |  |  |
| 12.805 | Phenol, 4-methoxy-3-methyl- |  |  |  |  |  | 1.07 |  |  |
| 12.869 | 2-Hexenoic acid, 3,4,4-trimethyl-5-oxo-, (Z)- |  |  |  |  |  | 1.35 |  |  |
| 12.889 | 1H-Pyrazole, 1,3,5-trimethyl- |  |  |  |  |  |  | 1.17 |  |
| 13.135 | Naphthalene, 1,2,3,4-tetrahydro- |  | 2.12 |  |  |  |  |  |  |
| 13.361 | 1H-Pyrrole, 3,4-diethyl-2-methyl- |  | 1.64 |  |  |  |  |  |  |
| 13.620 | Phenol, 3,5-dimethyl- |  | 1.21 |  |  |  |  | 2.16 |  |
| 13.866 | Phenol, 3-ethoxy- |  |  |  |  |  | 1.45 |  |  |
| 14.001 | 2,6-Dimethylbicyclo[3.2.1]octane |  |  |  |  |  | 1.49 |  |  |
| 14.131 | 1H-Pyrrole, 3-ethyl-2,4,5-trimethyl- |  |  |  |  |  | 1.69 | 2.15 | 3.40 |
| 14.209 | Phenol, 3-ethyl- |  |  |  |  |  |  | 1.72 |  |
| 14.254 | 3,4-Methylpropylsuccinimide | 1.54 |  | 3.02 | 1.34 | 2.88 |  |  |  |
| 14.422 | 2,5-Pyrrolidinedione, 3-ethyl-1,3-dimethyl- |  |  | 2.41 |  |  |  |  |  |
| 14.435 | 3-Methoxybenzylamine |  |  |  |  |  |  | 1.50 |  |
| 14.474 | 2H-Azepin-2-one, hexahydro-3-methyl- | 1.13 | 1.87 |  |  |  |  |  |  |
| 14.480 | Phenol, 3,5-dimethyl- |  |  |  |  | 1.46 |  |  | 2.65 |
| 14.855 | 9-Methylbicyclo[3.3.1]nonane |  |  |  |  |  | 1.47 |  |  |
| 14.894 | Benzenamine, 4-propoxy- |  | 1.24 |  |  | 1.87 |  |  |  |
| 15.269 | 1,4-Benzenediol, 2,3,5-trimethyl- |  |  |  |  |  | 1.66 |  |  |
| 15.276 | 2,4(1H,3H)-Pyrimidinedione, 5-amino- |  |  | 1.18 |  |  |  |  |  |
| 15.289 | Acetaldehyde, (3,3-dimethylcyclohexylidene)-, |  |  |  |  | 1.29 |  |  |  |
| 15.386 | Piperidine, 3-dimethylamino-1-methyl- |  |  |  |  |  |  | 1.78 |  |
| 15.418 | 3-Piperidinone, 1-ethyl-6-methyl- | 2.49 |  | 2.55 |  | 3.31 |  |  |  |
| 15.425 | 1-Butanamine, 2-methyl-N-(2-methylbutylidene)- |  | 2.58 |  | 1.35 |  |  |  |  |
| 15.444 | 1,2-Dimethyl-4-oxocyclohex-2-enecarboxaldehyde |  |  |  |  |  | 1.12 |  |  |
| 15.554 | N-(2-Propynyl)pyrrolidine |  | 1.14 |  |  |  |  |  |  |
| 15.632 | 3,4-Dimethylbenzyl alcohol |  |  |  |  | 2.25 |  |  |  |
| 15.638 | Phenol, 3-(ethylamino)-4-methyl- |  | 4.27 |  |  |  |  |  |  |
| 15.742 | Phenol, 3-(ethylamino)-4-methyl- |  |  | 1.49 |  |  |  | 1.71 |  |
| 16.039 | 1,5-Benzothiazepine, 2,3,4,5-tetrahydro-2,2,4-trimethyl- |  |  |  |  |  | 1.39 |  |  |
| 16.291 | N-[2-Hydroxyethyl]succinimide | 1.53 |  |  |  |  |  |  |  |
| 16.628 | 4,5-Dimethyl-ortho-phenylenediamine |  |  |  |  | 1.46 |  |  |  |
| 16.634 | 4-Hydroxy-3-methylbenzaldehyde |  | 1.04 |  |  |  |  |  |  |
| 17.016 | 4-Acetylaminomethylpyridine |  |  |  |  | 1.71 |  |  |  |
| 17.035 | 2,5-Dimethyl-3-isopropylpyrazine |  | 1.11 |  |  |  |  |  |  |
| 17.145 | Pyrazine, 5-butyl-2,3-dimethyl- |  |  |  |  |  |  |  | 1.48 |
| 17.611 | 10,10-Dimethyl-2,6-dimethylenebicyclo[7.2.0]undecan-5.beta.-ol |  |  |  |  |  | 1.28 |  |  |
| 17.656 | Benzaldehyde, 4-ethoxy- |  |  |  |  | 1.57 |  |  |  |
| 17.740 | Benzylcarbamate |  |  |  |  | 1.27 |  | 0.72 |  |
| 17.773 | Borazine, 2,4,6-triethyl- |  |  |  |  |  | 1.03 |  |  |
| 17.786 | Ethyltetramethylcyclopentadiene |  | 1.20 |  |  |  |  |  |  |
| 18.167 | Benzene, 1-(1,1-dimethylethoxy)-3-methyl- |  |  |  |  |  |  | 1.09 |  |
| 18.193 | N-(1-Cyclohexen-1-yl)piperidine |  |  |  |  |  | 1.47 |  |  |
| 18.213 | Piperidine, 1-methyl- |  |  | 4.33 |  |  |  |  |  |
| 18.245 | 1-Methyl-2-piperidinemethanol |  |  |  |  |  |  | 2.00 |  |
| 18.258 | 2-Pyridineacetic acid, hexahydro-1-methyl- |  |  |  |  | 4.43 |  |  |  |
| 18.271 | Mepivacaine |  | 6.68 |  |  |  |  |  |  |
| 18.290 | 2-Pyrrolidinone, 1-butyl- | 3.15 |  |  |  |  |  |  |  |
| 18.303 | (R)-1-Ethyl-2-pyrrolidinecarboxamide |  |  |  | 2.14 |  | 1.61 |  |  |
| 18.387 | Piperidine, 1-methyl- |  |  |  |  |  |  |  | 1.48 |
| 18.394 | Naphthalene, 1,2,3,4-tetrahydro-1,1,6-trimethyl- | 1.85 |  | 1.41 |  |  |  |  |  |
| 18.510 | 1-Methyl-4(axial)ethynyl-trans-decahydroquinolin-4(equat)-ol |  | 1.18 |  |  |  |  |  |  |
| 18.562 | 5,6,7,8-Tetrahydro-1,2,4-benzotriazine-3-amine |  | 1.20 |  |  |  |  |  |  |
| 19.164 | 5H-Inden-5-one, 1,2,3,6,7,7a-hexahydro-7a-methyl- |  | 1.01 |  |  |  |  |  |  |
| 19.170 | Acetamide, N-[1-(4-hydroxyphenyl)ethyl]- |  |  |  |  | 1.77 |  |  |  |
| 19.338 | Phenol, 3,5-dimethyl-, methylcarbamate |  |  |  |  | 1.29 |  |  |  |
| 19.694 | 2(3H)-Naphthalenone, 4,4a,5,6,7,8-hexahydro- |  |  | 1.45 |  |  |  |  |  |
| 19.830 | 1H-Indole, 3-methyl- |  | 1.13 | 0.72 |  |  |  |  |  |
| 20.968 | n-Dodecanoylpyrrolidine |  |  | 1.4 |  |  |  |  |  |
| 20.975 | Oxalic acid, 2-isopropylphenyl undecyl ester |  | 1.29 |  |  |  |  |  |  |
| 20.981 | Decane, 2-methyl- | 1.28 |  |  |  |  |  |  | 1.43 |
| 21.563 | N-Butyl-N-propyl-1-butanamine |  |  | 1.05 |  |  |  |  |  |
| 21.732 | 1-Pentadecene |  |  |  |  |  |  | 1.01 | 1.84 |
| 21.913 | Pentadecane | 1.12 |  |  |  |  |  |  | 1.36 |
| 22.036 | 2,6-Decadien-1-ol, 7-methyl-3-propyl- |  |  |  |  |  |  | 1.00 |  |
| 22.081 | Benzenamine, N-(1-methyl-2-propynyl)- |  | 1.37 | 1.34 |  | 1.31 |  |  |  |
| 22.288 | 1H-Indole, 2,3-dimethyl- |  |  |  |  |  |  | 1.45 |  |
| 24.054 | 1H-Indole, 5,6,7-trimethyl- | 1.19 |  |  |  | 1.05 |  |  |  |
| 27.068 | 8-Heptadecene |  |  |  |  |  |  |  | 1.74 |
| 27.120 | 2,3,7-Trimethylindole |  |  |  |  |  |  |  |  |
| 27.476 | Heptadecane | 1.43 |  | 2.57 |  |  |  |  |  |
| 27.631 | Dodecane, 4,6-dimethyl- | 1.07 | 1.28 |  |  |  |  |  |  |
| 28.588 | 1,7-Trimethylene-2,3,5-trimethylindole |  | 1.07 |  |  |  |  |  |  |
| 28.983 | N-Dinitrophenyl-l-tryptophane |  |  | 1.05 |  |  |  |  |  |
| 29.669 | Acetic acid, 3,7,11,15-tetramethyl-hexadecyl ester |  |  |  |  |  |  |  | 1.33 |
| 29.675 | 2-Hexadecene, 2,6,10,14-tetramethyl- | 1.69 | 1.25 |  |  |  |  |  |  |
| 30.180 | Hexadecane, 2,6,10,14-tetramethyl- | 8.24 | 8.26 | 3.04 |  | 2.21 |  |  |  |
| 30.568 | 2-Hexadecene, 3,7,11,15-tetramethyl-, | 8.55 | 9.80 | 1.93 |  | 5.23 |  | 2.38 | 2.38 |
| 31.027 | Cyclododecanone, 2-methylene- |  |  |  |  |  |  |  | 3.37 |
| 32.308 | Pentadecanenitrile | 2.09 |  |  | 6.26 |  |  |  |  |
| 37.884 | Hexadecanamide | 15.91 | 2.74 | 6.56 | 36.9 | 3.38 |  | 4.30 | 1.39 |
| 38.440 | N-Methyldodecanamide |  |  | 3.39 | 18.2 |  |  |  |  |
| 38.447 | 1,12-Dodecanediamine | 5.18 |  |  |  |  |  | 1.34 |  |
| 38.453 | Hexanal, O-methyloxime |  | 3.95 |  |  | 1.79 |  |  |  |
| 39.165 | Octanamide, N,N-dimethyl- |  |  | 1.39 |  |  |  |  |  |
| 39.171 | N,N-Dimethyldodecanamide | 1.38 |  |  | 8.35 |  |  |  |  |
| 40.717 | Succinic acid, nonyl 2,3,5-trichlorophenyl ester |  |  |  | 2.83 |  |  |  |  |
| 41.066 | 9-Octadecenamide, (Z)- | 12.69 |  |  |  |  |  |  |  |
| 41.150 | Octadecanamide |  |  | 1.75 |  |  |  |  |  |
| 41.467 | Dodecanamide, N,N-diethyl- | 2.35 |  | 1.66 |  |  |  |  |  |
| 41.474 | Octadecanamide, N-butyl- |  | 1.14 |  | 4.70 |  |  |  |  |
| 41.564 | Phenol, 6-methyl-2-[(4-morpholinyl)methyl]- | 1.21 |  |  |  |  |  |  |  |
| 43.156 | 5-Methyl-2-phenylindolizine | 1.90 |  | 2.29 | 5.10 |  |  |  |  |
| Total | | 75.4 | 62.9 | 91.9 | 80.2 | 83.1 | 56.8 | 96.6 | 83.1 |

*peak area at least 1.0% of the total peak area
