# Supplementary material for: Liquid fuel generation from algal biomass via a two-step process: effect of feedstocks
Source: Biotechnol Biofuels. 2018 Apr 2;11:83. doi: 10.1186/s13068-018-1083-2 (PMC5879921; doi:10.1186/s13068-018-1083-2)
Supplement: Supplementary file 2 — Additional file 2: Figure S1. Total ion chromatograms of upgraded bio-oils produced from the upgrading of eight different crude bio-oils. [file 13068_2018_1083_MOESM2_ESM.docx]

12

**Figure S1.** Total ion chromatograms of upgraded bio-oils produced from the upgrading of eight different crude bio-oils
